# Outcomes of transarterial chemoembolization in patients with unresectable hepatocellular carcinoma: A tertiary center experience

**DOI:** 10.12669/pjms.40.6.7593

**Published:** 2024-07

**Authors:** Misbah Tahir, Khalid Mustafa, Muhammad Ali, Danial Khalid

**Affiliations:** 1 Misbah Tahir, FCPS Department of Radiology, Liaquat National Hospital, National Stadium Road, Karachi, Pakistan; 2 Khalid Mustafa, FCPS Department of Radiology, Liaquat National Hospital, National Stadium Road, Karachi, Pakistan; 3 Muhammad Ali, FCPS Department of Radiology, Liaquat National Hospital, National Stadium Road, Karachi, Pakistan; 4 Danial Khalid, FCPS Department of Radiology, Liaquat National Hospital, National Stadium Road, Karachi, Pakistan

**Keywords:** Hepatocellular carcinoma, Trans-arterial chemoembolization (TACE), Median survival time, Unresectable HCC

## Abstract

**Objective:**

To assess the overall survival in patients with intermediate stage hepatocellular carcinoma following transarterial chemoembolization.

**Methods:**

It is a retrospective descriptive study carried out in the Department of Radiology of Liaquat National Hospital Karachi, Pakistan. Seventy-two patients were enrolled from July 2014 to December 2021 and had chemoembolization therapy. Patients were followed till their demise. Mean and Median survivals were calculated.

**Results:**

A total of 72 patients had a median survival of 15 months with 95% confidence interval (11 months was lower bound and 18 months was upper bound), 19 months was the mean survival time with 95% confidence interval (14.7 months was lower limit and 22.6 months the upper limit). The factors which had a significant impact on the median survival time were Child-Pugh classification, average size of tumor and embolization pattern.

**Conclusion:**

Transarterial chemoembolization (TACE) increases the median survival time effectively and safely in patients with hepatocellular carcinoma. However complete resolution of disease is not possible with TACE, with most patient eventually succumbing to the disease. The overall survival for TACE in this study correlates well with other studies. Child Pugh Class, tumor size and embolization pattern have significant effect on survival of patients.

## INTRODUCTION

Hepatocellular carcinoma (HCC) is ranked third in the causes of deaths related to cancer globally.^1^ HCC predominantly occurs in patients with chronic liver disease which in Pakistan is mainly due to hepatitis C and hepatitis B virus infection.^2^ Having a poor prognosis, the overall five-year survival of HCC is less than 20%. The individual survival of patients is determined by disease stage.^3^ Liver transplantation, surgical resection and microwave or radiofrequency ablation are treatment options^4^,^5^ for patients with limited disease. Liver transplantation has shown good outcomes, with a five years survival of 70% and a 10 years survival of 50%.^6^ However due to financial constraints and scarcity of trained hepatic surgeons, transplantation is not readily available. Also, many patients present late with intermediate stage disease which precludes transplantation, surgical resection and ablation. TACE either alone or in combination with ablation becomes the main stay of treatment. For patients with intermediate-stage HCC, the Barcelona Clinic Liver Cancer (BCLC) staging system and other guidelines recommend TACE as the primary choice, although TACE has been used for other indications.^7^

Although TACE prolongs survival it becomes less effective with each subsequent treatment.^8^ Various cytotoxic drugs have been used for TACE such as epirubicin, doxorubicin, fluorouracil and cisplatin. These drugs are given along with Lipiodol, an iodinated oily contrast agent, which enhances uptake of the drugs into the tumor. As the tumor is primarily supplied by arteries, the arteries are selectively cannulated and the drugs are slowly given into the tumor. TACE exerts its effect through both ischemic and cytotoxic processes on the HCC, with ischemia having a greater role.^9^ Some have also used PVA (polyvinyl alcohol) particles in place of Lipiodol with good results. It has been proven by various studies that TACE is effective in prolonging patient survival, particularly in patients with intermediate stage hepatocellular carcinoma. In a study by Yang et al., the median overall survival in TACE responsive HCC was 34 months following TACE, whereas it was 21 months in non-responsive tumors.^10^ Another study showed the median survival time to be 37 months following first session of TACE.^11^

Trans-arterial radioembolisation (TARE) is a further advancement in TACE which is gaining popularity. However, being expensive and without showing a great deal of advantage in terms of survival over TACE, TACE still remains the popular choice in places with limited resources. A research comparing conventional TACE with TARE showed the median survival time to be 17.7 months for conventional TACE vs 18.6 months for TARE.^12^ Patient survival, improvement in quality of life and symptoms are the most significant factors in assessment of efficacy of TACE. We assessed the efficacy of TACE by measuring its effect on survival of patients with unresectable HCC.

**Fig.1:**
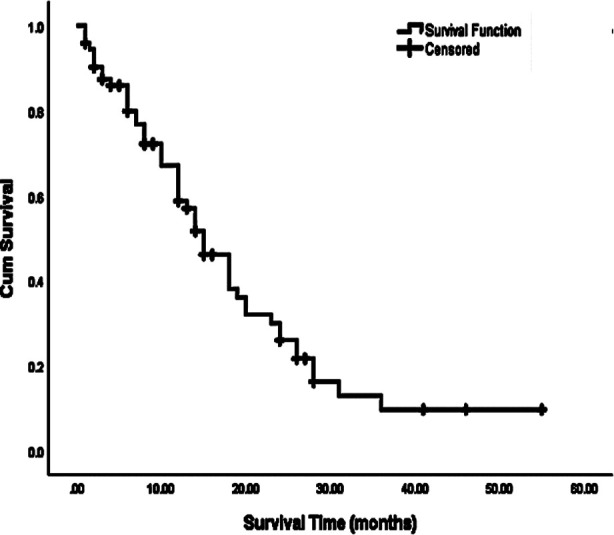
Overall survival time in months of patients having HCC after TACE.

## METHODS

It is a retrospective descriptive study carried out in the Department of Radiology of Liaquat National Hospital Karachi, Pakistan from February 2022 to December 2022.

The archives of the angiography suite were used to search patients with unresectable HCC who had undergone TACE between July 2014 to December 2021. Non probability (nonrandom) convenient sampling technique was used in our study. Patient data was collected from medical and radiological records. Laboratory findings like liver function tests, prothrombin time, serum albumin, serum alpha fetoprotein and imaging findings of MRI or triphasic CT scan of abdomen were noted before and after TACE and in follow ups. Child-Pugh classification as described by Pugh et al.^13^ was noted for every patient. Patients with complete record of TACE, laboratory reports, imaging findings and follow ups were included in study. Patients who were lost to follow up or had treatment other than TACE were excluded. Vascular involvement, number, extent, size and enhancement pattern of tumors were assessed in pre and post TACE CT scans and in sequential follow ups. First follow up was done after six weeks, then after three months, six months and then yearly.

### Ethical Approval:

Ethics review committee approval was received on February 2, 2022 having Ref: App # 0737-2021 LNH – ERC.

Criteria for repeating TACE were residual tumor enhancement, appearance of new lesion or growth in size of previously treated lesion. Demographic details (age and gender) were obtained. The presence of portal vein tumor thrombus (PVT) was determined by CT scan.^14^ Size of tumor was recorded in three categories i.e., <5cm, 5-7cm and >7cm. TACE was performed by selectively cannulating the arteries supplying the tumor with micro catheter and embolizing with emulsion made with 50mg Epirubicin and 10mL lipiodal. Poly vinyl alcohol particles (150 to 300 μm) were used where necessary. Mean and median survival time were calculated via Kaplan Meier method with 95% confidence interval.

## RESULTS

Seventy-two patients who had undergone TACE for HCC were included in the study which comprised of 49 males and 23 females with age range of 35-81 years. Ten patients had biopsy proven HCC and 62 patients had characteristic imaging findings of HCC. Ten patients had Hepatitis-B, 57 had Hepatitis-C, two had both Hepatitis-B and C. Three patients were seronegative. Total of 130 sessions were done in 72 patients. TACE was done for 129 lesions in 72 patients.

All patients had one to four tumors with a mean of 1.8. Fifteen months was the median survival time with 95% confidence interval (10.84 months lower bound and 17.75 months upper bound). Nineteen months was mean survival time with 95% confidence interval (14.79 months was lower bound and 22.68 months was upper bound).

HCC measured less than 5cm in 20 patients, 5-7cm in 17 patients and more than 7cm in 35 patients. Significant difference was noted in median and mean survival with regard to tumor size. Median survival time was 26 months for patients with tumor size less than 5cm, 20 months for tumor size of 5-7 cm and eight months for tumor size greater than 7cm. (Table-I)

**Table-I:** Tumor size and median survival time .

Item	Subgroups	N	OS (months)			OS (months)		

			Mean	95% CI	p	Median	95% CI	P
Average size of tumor	<5 cm	20	28	21-36	0.001	26	17-35	0.001
	5-7 cm	17	20	17-23		20	15-25	
	>7 cm	35	8	2-15		8	12-18	

Child-Pugh score affected the mean survival time significantly with a p-value of 0.001(c2 = 73.89; df =2). Thirty-seven patients were categorized as Child class A, 28 as B and 7 as C. Median survival decreased with worsening of Child class with p-value of 0.001. Child class A patients showed a median survival of 19 months, whereas it was 12 months for Child class B and two months for Child class C (Table-II). Out of 72 patients 40 had complete embolization and 32 had partial embolization.

**Table-II:** Child-Pugh class and median survival time.

Item	Subgroups	N	OS (months)			OS (months)		

			Mean	95% CI	p	Median	95% CI	P
Child-Pugh Class	A	37	24	18-30	0.001	19	13-25	0.001
	B	28	14	11-18		12	6-18	
	C	7	2	1-2		2		

Analysis showed a significant difference in median survival time with p value of 0.002, df=1, C2 = 9.21. The median survival time was 19 months in patients with complete embolization and 12 months in patients with partial embolization. (Table-III) Age, gender, hepatitis types, lobe of liver, portal vein thrombosis had no significant impact on overall survival (OS).

**Table-III:** Pattern of embolization and median survival time.

Item	Subgroups	N	OS (months)			OS (months)		

			Mean	95% CI	P	Median	95% CI	p
Embolization	Complete	40	24	18-31	0.002	19	11-27	0.002
	Partial	32	13	10-15		12	8-16	

## DISCUSSION

Effectiveness of TACE has been established in several case control and review studies. Though TACE has been compared with systemic therapy^15^ in randomized controlled trials, no comparison has been made with untreated patients as control subjects. The median survival in a study conducted in Aga Khan University Hospital was 13.6 months compared to 15 months in our study.^16^ A study conducted in China, showed one and two years survival rates of patients treated with TACE to be 56.49% and 18.83%, respectively. The mean survival time was 9.0 months.^17^ The reason that study showed poor survivals in comparison to our study is that they included all patients with portal vein thrombosis.

In an Egyptian Study^18^ the median survival time was 16 months, which is comparable to ours. They did not include Child C patients however, whereas in our study there were seven. Nor did they include patients with portal vein thrombosis whereas in our study there were thirteen. In a Chinese study carried out by Song et al, median survival time was 10.3 months.^19^ The relatively poor results may be due to the fact that they included many patients having portal vein thrombosis in their study. Their survival results correspond to the previously stated research^17^ which included subjects with portal vein thrombosis.

A meta-analysis carried out in USA showed median survival of 19.4 months.^20^ A study conducted in China compared survival between those who received TACE and those who were treated conservatively. Median survival of eight months was seen in patients who underwent TACE and two months who did not.^21^ The poor survival in their study was probably because all their patient had advanced disease or relatively poor functional status. Another study in which PVA particles and cisplatin were used showed median overall survival of 16.3 months.^22^ Another research showed the median survival time to be 17.7 months for TACE.^12^

Our study showed that underlying liver disease, tumor size and embolization pattern affected survival significantly. Patients with severe disease (Childs C) had poor prognosis (two months) compared to patients with Child A (19 months). In our study the median survival of patients with tumor size less than 5cm was 25.6 months which was better than patients having tumor size greater than 5cm (20 months). Further decrease in median survival was seen with tumor size greater than 7cm (8 months) as shown in Table-I. Our result is supported by another study which showed five-year survival in patients with tumor size less than 5cm to be 72.4% compared to patients with tumor size greater than 5cm of 32.3%.^23^

In our study patients with complete embolization had better survival than those with partial embolization as seen in Table-III. Hence complete embolization should be attempted balancing it with patient safety. As the chemotherapeutic agents are exclusively delivered to the tumor, TACE has minimal systemic side effects. For safety we restricted ourselves to 50 mg of Epirubicin which is the lower limit of allowed chemotherapeutic dose. There was no procedure related death in any patient and only a small number of patients developed post-embolization syndrome which was transient and was treated successfully by conservative management.

Research in which patients with hepatocellular carcinomas and portal vein tumor thrombus were treated with TACE using drug eluting beads showed the median overall survival time to be 14 months.^24^ As similar survival time was seen in studies in which conventional TACE was performed including this one, it can be inferred that using drug eluting beads confers only a limited advantage over conventional TACE. Another Chinese research showed overall survival of 21.0 months for patients with intermediate stage HCC who had undergone TACE.^25^ Overall the median survival time in our study correlates well with other studies.

### Limitations:

With a small sample size, the current study examined the experiences of one center in Karachi, Pakistan. As a result, conclusions could not be applied to all Pakistani patients. To validate the results of the current study, we propose conducting a broader investigation.

## CONCLUSION

TACE increases the median survival time effectively and safely. However complete resolution of disease is not possible with TACE with most patient eventually succumbing to the disease. The overall survival for TACE in this study correlates well with other studies. Child-Pugh Class, tumor size and embolization pattern have significant effect on survival of patients.
